# Relaxed skin tension line-oriented keystone-designed perforator island flaps considering the facial aesthetic unit concept for the coverage of small to moderate facial defects

**DOI:** 10.1097/MD.0000000000014167

**Published:** 2019-01-18

**Authors:** Chi Sun Yoon, Hyo Bong Kim, Young Keun Kim, Hoon Kim, Kyu Nam Kim

**Affiliations:** a Department of Plastic and Reconstructive Surgery, Ulsan University Hospital, University of Ulsan College of Medicine, Ulsan; b Department of Plastic and Reconstructive Surgery, Konyang University Hospital, University of Konyang College of Medicine, Myunggok Medical Research Center; c Kim Young Keun's Plastic and Aesthetic Surgery Clinics, Daejeon, Korea.

**Keywords:** face, plastic, reconstructive surgical procedures, surgical flaps

## Abstract

A variety of reconstructive options including skin grafts, loco-regional flaps, and free flaps exist for the coverage of facial defects. Each technique has advantages and disadvantages. In this study, we present our experience with relaxed skin tension line (RSTL)-oriented, keystone-designed perforator island flap (KDPIF) reconstructions, in consideration of the facial aesthetic unit concept for coverage of small to moderate facial defects.

Between May 2016 and February 2018, 17 patients (11 men and 6 women), with an average age of 63.53 years (range: 37–83 years) underwent KDPIF reconstructions to cover facial defects. We performed the KDPIF reconstructions in consideration of the RSTLs and facial aesthetic unit concept. We retrospectively reviewed the defect causes, locations, and sizes, and flap sizes, types, and survivals, as well as the complications. Cosmetic outcomes were evaluated using the Harris 4-stage scale from 3 independent plastic surgeons’ point of view, and postoperative satisfactory surveys from all patients.

All defects were successfully covered with KDPIF. The defect sizes varied from 1.5 × 1.5 cm^2^ to 3 × 3.5 cm^2^. The flap sizes varied from 1.5 × 3 cm^2^ to 3 × 5.5 cm^2^. All flaps fully survived and there were no postoperative complications. The average subjective patient satisfaction score was 8.29 (range: 7–10) and the objective cosmetic outcomes were favorable (fair, good, or excellent).

Considering its simplicity and safety, the KDPIF is a good reconstruction option with few complications and high reproducibility. The RSTL-oriented KDPIF reconstruction is a good reconstructive option for covering small to moderate facial defects with superior aesthetic outcomes.

## Abstract 2

Supplemental Digital Content is available in the text

## 1 Introduction

Procedures to cover facial defects are common in the field of plastic and reconstructive surgery. Although defects are rarely life-threatening and defect sizes are not extensive, the reconstruction of facial defects can be complex and significantly impact a patient's facial function and aesthetics.^[1]^ A variety of reconstructive options are available including skin grafts, loco-regional flaps, and free flaps for the repair of facial defects. Because each technique has advantages and disadvantages, reconstructive surgeons choose the appropriate method on a case-by-case basis.^[2]^ Skin grafts, especially full-thickness skin grafts of the face are easily and readily performed. However, the surgical outcomes can be limited in cases affected by contour irregularities, mismatches of color and texture, and donor site morbidities. Skin grafting cannot be performed when wounds are infected or when vital structures (e.g., major vessels and bony structures) are exposed.^[2]^ Thus, skin grafts should be indicated for partial thickness defects with intact underlying musculatures. Free tissue transfers are a good option for larger facial defects; however, they may reflect overtreatment for smaller facial defects. Additionally, the usefulness of these transfers can be limited by the lack of skilled microsurgeons, the inability of centers to perform postoperative microsurgical monitoring and care, and the presence of comorbidities that prohibit lengthy operations.^[2]^ Local flaps are considered the best reconstructive option for small- to moderate-sized defects of the face. Because these flaps are harvested from adjacent tissues, they provide good matches in terms of color and texture, which is consistent with the ideal goals of reconstruction (like-with-like) of the face. Various local flaps, such as random pattern flaps (e.g., rhomboid flaps, bilobed flaps, modified Limberg flaps, and V-Y advancement flaps), axial flaps, local perforator flaps, and keystone-designed perforator island flaps (KDPIF) have been used for facial defect coverage.

Among the flap options, the KDPIF, which were devised by Behan in 2003, have a curvilinear-shaped trapezoidal design and essentially comprise 2 end-to-side V-Y flaps.^[3]^ KDPIF have grown in clinical applicability in various fields of reconstructive surgery in the past decade.^[4–8]^ However, previous studies have only presented KDPIF reconstructions of facial defects in limited locations, such as large parotid defects and small-to-moderate nasal defects, but did not consider the facial aesthetic unit concept in detail. Therefore, to the best of our knowledge, we present the first report of, and a description of our experience with, relaxed skin tension line (RSTL)-oriented KDPIF reconstruction considering the facial aesthetic unit concept for the coverage of small-to-moderate facial defects in various regions.

## 2 Material and methods

Between May 2016 and February 2018, 17 patients (11 men and 6 women), with an average age of 63.53 years (range: 37–83 years) underwent KDPIF reconstructions to cover facial defects. We retrospectively reviewed the defect causes and locations based on the facial aesthetic unit concept, the defect sizes, flap sizes, types of KDPIF (type I: skin incision only; type IIA: division of the deep fascia along the outer curvilinear line; type IIB: division of the deep fascia with skin graft to the secondary defect; type III: opposing Keystone flaps designed to create a double-keystone flap; type IV: Keystone flap with undermining of up to 50% of the flap subfascially),^[3]^ flap survivals, complications, and the follow-up periods for each patient. All patients were asked to rate their subjective satisfaction with the postoperative outcome on a scale of 1 to 10 at the final follow-up.^[9]^ Three independent plastic surgeons graded the postoperative cosmetic outcomes (plastic surgeon's global impression of changes) as excellent, good, fair, or poor, according to the Harris 4-stage scale, by comparing the preoperative and postoperative clinical photographs.^[9,10]^

This study protocol was approved by the ethical review board of Konyang University Hospital (KUH) approval number (2018-07-007) and all patients provided written informed consent.

## 3 Surgical techniques

The operations were performed with the patients in the supine position under general or local anesthesia. After debridement or excision of the lesions, we performed the KDPIF reconstruction in consideration of RSTLs and the facial aesthetic unit concept. When designing the flap in accordance with the defect size, several points were considered. First, the width of the flap was designed to be either equal to or slightly larger than the width of the defect when 1 KDPIF was used (Type IIA KDPIF). When a Type III KDPIF (opposing Keystone flaps designed to create a double-keystone flap) was used, especially with larger defects and midline-crossing defects, the width of the flap was designed to be slightly smaller than the width of the defect. Second, the long axis of the flap was made parallel to the RSTLs as much as possible to minimize wound tension and scar formation. Third, the flap and the incisions were located within and along each facial aesthetic unit, which would allow for distraction from the final postoperative appearance, thus creating the illusion of normal skin architecture. Fourth, the Ω-variant KDPIF was performed in case of defects with less laxity of the surrounding tissue, which need more flap movement to achieve tension-less coverage; such modification provides additional flap movement such as rotation. Namely, if more tension reduction is required for wound closure, the Ω-variant KDPIF is more suitable than the original KDPIF in consideration of tissue laxity. Once the skin incision was made along the flap, dissection proceeded from the subcutaneous layer to the deep fascia (superficial muscular aponeurotic system [SMAS] layer). The fibrous subcutaneous septa and deep fascia were released using a monopolar device until the flap could be moved freely from the surrounding tissues. Then, care was taken to separate the circumferential tissues, with minimal undermining of the flap margin (3–4 mm from the flap margin in the present study) to preserve the integrity of the perforators. Unlike other local flaps such as the rotation flap, Limberg flap, and bilobed flap, minimal flap undermining with maximal preservation of perforator hot spots is characteristic of the KDPIF. The basic movement of the KDPIF is achieved by advancement via releasing the fibrous subcutaneous septa and deep fascia, and further movement can be acquired by minimal undermining of the flap margin. After meticulous efforts at hemostasis, re-approximating mattress sutures were placed to close the defect, with each end aligned in a V-Y apposition. The donor site was closed in a primary fashion and a mild compression dressing made of a foam material was placed.

## 4 Results

The patient characteristics and clinical data are summarized in Table 1. The causes of the defects included wide excisions of skin malignancies in 6 patients, trauma in 4, ruptured epidermal cyst excisions in 4, burns in 2, and a foreign body granuloma with an abscess in 1. The locations of the defects included the temporal area in 2 cases, the nose in 4, the cheek area in 4, the preauricular area in 1, the malar area in 3, the nasolabial fold in 1, the forehead in 1, and the angle of the mandible area in 1. The defect sizes varied from 1.5 × 1.5 cm^2^ to 3 × 3.5 cm^2^. All defects were successfully covered with KDPIF types as follows: Type IIA KDPIF was used in 13 patients, Ω-variant^[11]^ Type III KDPIF in 2, Ω-variant Type IIA KDPIF in 1, and Sydney melanoma unit (SMU) modification^[12]^ Type IIA KDPIF with Ω-variant KDPIF in 1. The flap sizes varied from 1.5 × 3 to 3 × 5.5 cm^2^. All flaps fully survived and there were no postoperative complications. The average subjective patient satisfaction score was 8.29 (range: 7–10) and all patients were fairly satisfied with their final aesthetic outcome after an average follow-up period of 8.24 months (range: 6–12 months). Postoperative cosmetic outcomes evaluated by 3 independent plastic surgeons were favorable overall (scale: fair, good, or excellent) (see Table S1, Supplemental Content, which illustrates the postoperative cosmetic outcomes, namely, plastic surgeon's global impression of changes according to the Harris 4-stage scale and postoperative patient-satisfaction surveys).

**Table 1:**
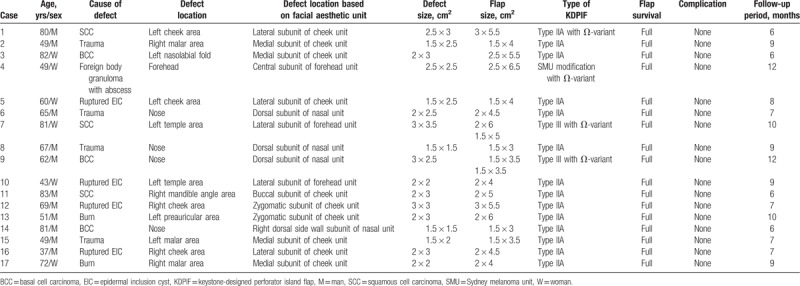
Patient data.

## 5 Case presentations

### 5.1 Case 1

**Figure 1:**
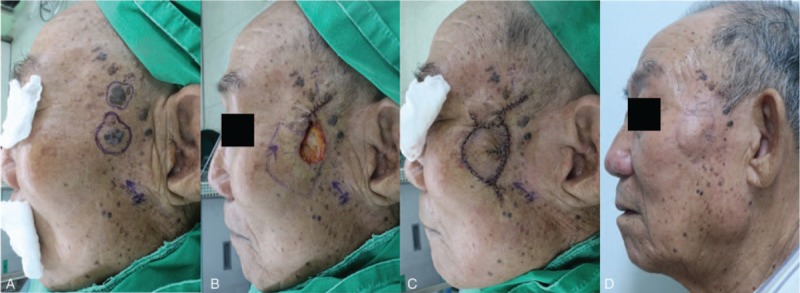
(A) An 80-year-old man was diagnosed with squamous cell carcinoma in the left cheek area (lateral subunit of the cheek unit). (B) He underwent a wide excision with a 4-mm safety margin and the final defect was measured to be 2.5 × 3 cm^2^. (C) We covered the defect with a Ω-variant Type IIA keystone-designed perforator island flap (flap size: 3 × 5.5 cm^2^) from the anterior side of the defect. (D) Postoperative clinical photograph after 6 months of follow-up.

An 80-year-old man was diagnosed with squamous cell carcinoma of the left cheek area (Fig. 1). We planned a wide excision with local flap coverage under local anesthesia. He underwent wide excision with a 4-mm safety margin, and the final defect size was 2.5 × 3 cm^2^ on the lateral subunit of the cheek unit in view of the facial aesthetic unit concept. We covered the defect with a 3 × 5.5 cm^2^-sized Ω-variant Type IIA KDPIF from the anterior side of the defect, in the light of the facial RSTL and the aesthetic unit concept. Both the insetting of the flap and the primary closure of the donor site were achieved without tension or drain placement. The flap survived completely without postoperative complications. The final appearance was evaluated after a 6-month follow-up period, and the patient was satisfied with the final outcome, providing a subjective satisfaction score of 8. The postoperative cosmetic outcome evaluated by 3 independent plastic surgeons was rated as good (2 raters) and excellent (1 rater).

### 5.2 Case 3

**Figure 2:**
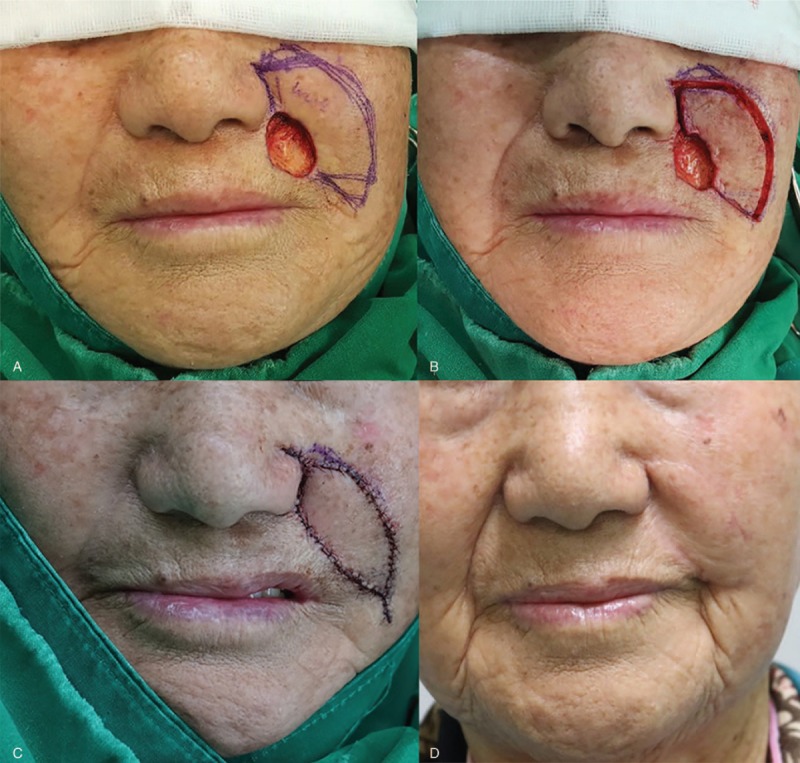
An 82-year-old woman was diagnosed with basal cell carcinoma in the left nasolabial fold area (medial subunit of the cheek unit) by punch biopsy. (A) She underwent a wide excision with a 4-mm safety margin and the final defect was measured to be 2 × 3 cm^2^. (B, C) We covered the defect with a Type IIA keystone-designed perforator island flap (flap size: 2.5 × 5.5 cm^2^) from the upper-lateral side of the defect. (D) Postoperative clinical photograph after 6 months of follow-up.

An 82-year-old woman was diagnosed with a basal cell carcinoma in the left nasolabial fold area after a punch biopsy (Fig. 2). We planned a wide excision with local flap coverage under local anesthesia. She underwent wide excision with a 4-mm safety margin and the final defect size was 2 × 3 cm^2^ on the medial subunit of the cheek unit, in view of the facial aesthetic unit concept. We covered the defect with a 2 × 5.5 cm^2^-sized Type IIA KDPIF from the upper-lateral side of the defect, in the light of the facial RSTL and the aesthetic unit concept. Both the insetting of the flap and the primary closure of the donor site were achieved without tension or drain placement. The flap survived completely without postoperative complications. No tumor recurrence was observed during the 6-month follow-up period, and the patient was satisfied with the final outcome, providing a subjective satisfaction score of 9. The postoperative cosmetic outcome evaluated by 3 independent plastic surgeons was rated as excellent (2 raters) and good (1 rater).

### 5.3 Case 7

**Figure 3:**
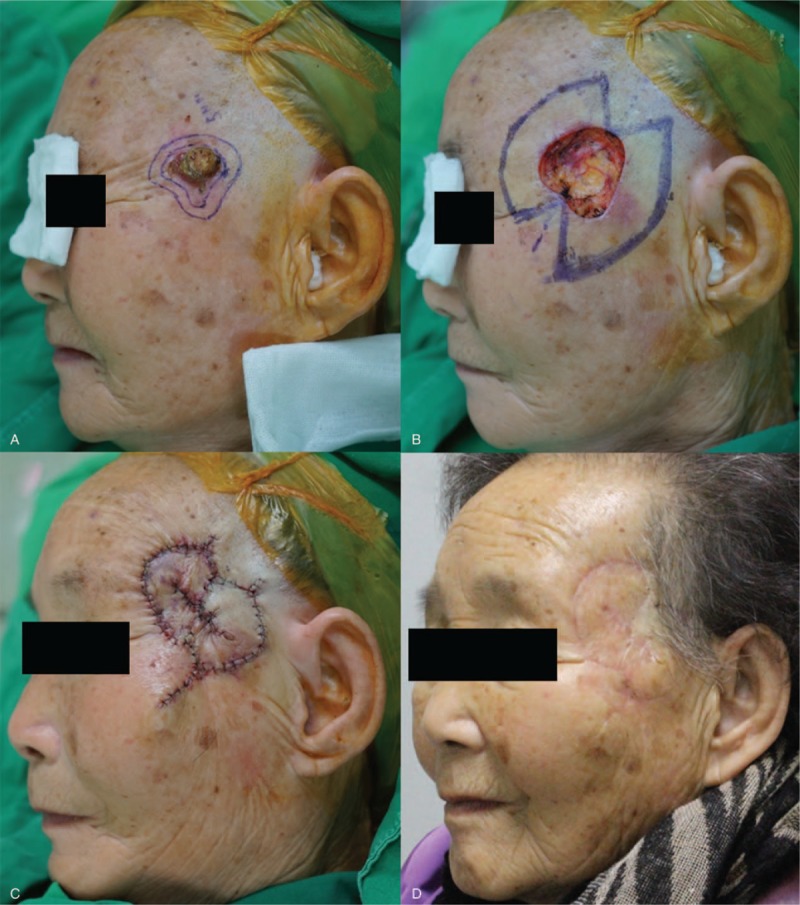
An 81-year-old woman was diagnosed with squamous cell carcinoma in the left temporal area (lateral subunit of the forehead unit) after a punch biopsy. (A, B) She underwent a wide excision with a 5-mm safety margin, and the final defect was measured to be 3 × 3.5 cm^2^. (C) We covered the defect with bilateral Ω-variant keystone-designed perforator island flaps (flap sizes: 2 × 6 cm^2^ and 1.5 × 5 cm^2^). (D) Postoperative clinical photograph after 10 months of follow-up.

An 81-year-old woman was diagnosed with squamous cell carcinoma in the temporal region after a punch biopsy (Fig. 3). We planned a wide excision with local flap coverage under local anesthesia. She underwent wide excision with a 5-mm safety margin and the final defect size was 3 × 3.5 cm^2^ on the lateral subunit of the forehead unit in view of the facial aesthetic unit concept. We covered the defect with an Ω-variant Type III KDPIF, wherein the sizes of the medial and lateral flaps were 1.5 × 5 cm^2^ and 2 × 6 cm^2^, respectively, in light of the facial RSTL and the aesthetic subunit concept. Both the insetting of the flap and the primary closure of the donor site were achieved without tension or drain placement. The flap survived completely without postoperative complications. No tumor recurrence was observed during the 10-month follow-up period, and the patient was satisfied with the final outcome, providing a subjective satisfaction score of 9. The postoperative cosmetic outcome evaluated by 3 independent plastic surgeons was rated as excellent (2 raters) and good (1 rater).

### 5.4 Case 9

**Figure 4:**
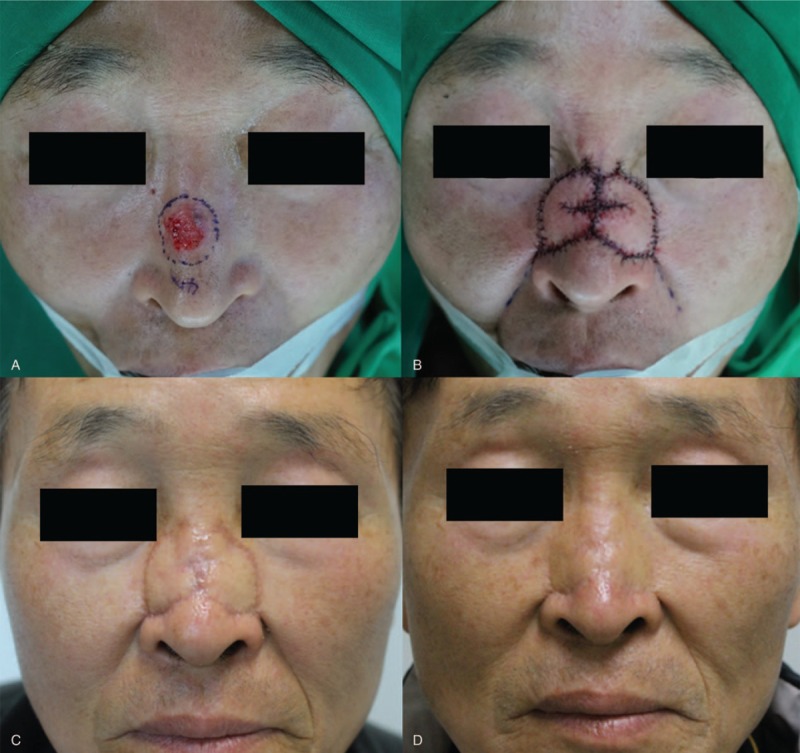
A 62-year-old man was diagnosed with basal cell carcinoma on the nose (dorsal subunit of the nasal unit) after a punch biopsy. (A) He underwent wide excision with a 4-mm safety margin, and the final defect was measured to be 3 × 2.5 cm^2^. (B) We covered the defect with bilateral Ω-variant keystone-designed perforator island flaps (each flap size was 1.5 × 3.5 cm^2^) from both dorsal side wall subunits. (C) Postoperative clinical photograph after 1 month of follow-up. (D) Postoperative clinical photograph after 12 months of follow-up.

A 62-year-old man was diagnosed with basal cell carcinoma of the nose after a punch biopsy (Fig. 4). We planned a wide excision with local flap coverage under general anesthesia. He underwent wide excision with a 4-mm safety margin and the final defect size was 3 × 2.5 cm^2^ on the dorsal subunit of the nose, in view of the facial aesthetic unit concept. We covered the defect with an Ω-variant Type III KDPIF, with a flap size of 1.5 × 3.5 cm^2^ from each dorsal side wall subunit, in light of the facial RSTL and the aesthetic unit concept. Both the insetting of the flap and the primary closure of the donor site were achieved without tension or drain placement. The flap survived completely without postoperative complications. No tumor recurrence was observed during the 12-month follow-up period, and the patient was satisfied with the final outcome, providing a subjective satisfaction score of 10. The postoperative cosmetic outcome evaluated by 3 independent plastic surgeons was rated as excellent by all the raters.

## 6 Discussion

The KDPIF technique is advantageous with respect to its simple defect-adaptive design, easy reproducibility, safety, and short procedure time due to minimal flap undermining and dissection. KDPIF achieves an ideal reconstruction by replacing “like-with-like” without difficulty.^[3–7,13,14]^ In this study, we found that the RSTL-oriented KDPIF reconstruction, considering the facial aesthetic subunit concept, may be a sound alternative to conventional local and perforator flaps for covering small-to-moderate facial defects. Additionally, the RSTL-oriented KDPIF reconstruction considering the facial aesthetic subunit concept achieves superior aesthetic outcomes.

**Figure 5:**
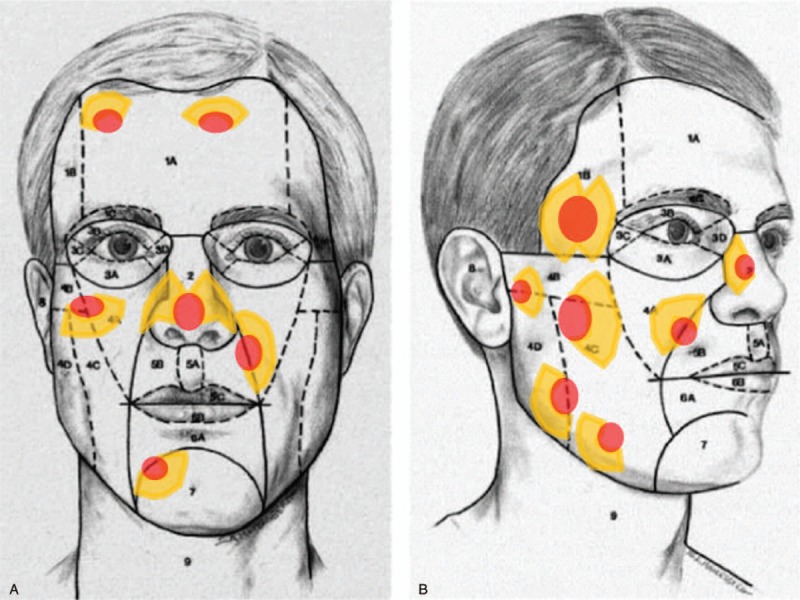
Schematic diagram showing the relaxed skin tension line-oriented KDPIF considering the facial aesthetic unit concept. Red-colored ellipses represent defects and yellow-colored figures represent the design of KDPIF. (A) Frontal and (B) profile views of the aesthetic units and subunits of the face. 1, Forehead unit (1A, central subunit; 1B, lateral subunit; 1C, eyebrow subunit); 2, nasal unit; 3, eyelid units (3A, lower-lid unit; 3B, upper-lid unit; 3C, lateral canthal subunit; 3D, medial canthal subunit); 4, cheek unit (4A, medial subunit; 4B, zygomatic subunit; 4C, lateral subunit; 4D, buccal subunit); 5, upper-lip unit (5A, philtrum subunit; 5B, lateral subunit; 5C, mucosal subunit); 6, lower-lip unit (6A, central subunit; 6B, mucosal subunit); 7, mental unit; 8, auricular unit; 9, neck unit. (Reprinted from Fattahi^[16]^, with permission from Elsevier.). KDPIF = keystone-designed perforator island flaps.

**Figure 6:**
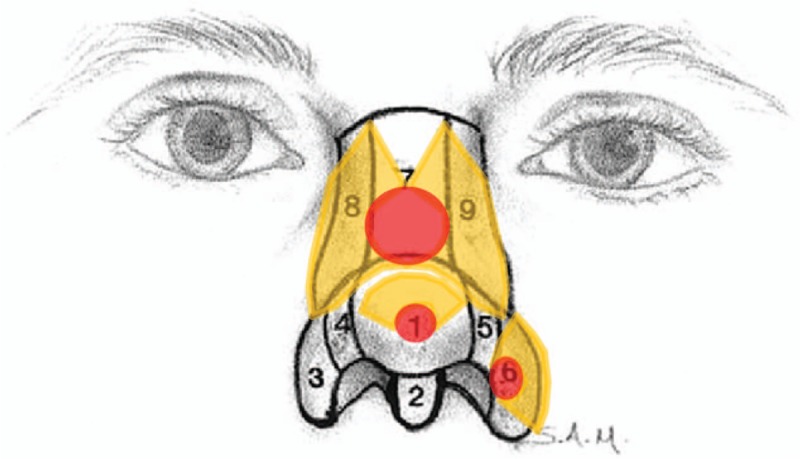
Schematic diagram showing the relaxed skin tension line-oriented KDPIF considering the nasal unit. Red-colored ellipses represent defects and yellow-colored figures represent the design of KDPIF. 1, Tip subunit; 2, columellar subunit; 3, 6, right and left alar base subunits; 4, 5, right and left alar side wall subunits; 7, dorsal subunit; 8, 9, right and left dorsal side wall subunits. (Reprinted from Fattahi^[16]^, with permission from Elsevier.). KDPIF = keystone-designed perforator island flaps.

Facial skin and soft tissue defects are some of the most common problems that plastic and reconstructive surgeons encounter. A large variety of reconstructive methods have been developed and used. The local flap technique may be the most suitable modality for covering small- to moderate-sized full-thickness defects of the face. Local flaps can readily achieve ideal reconstruction goals (like-with-like). Fundamentally, to obtain better aesthetic and functional results in facial reconstructions using local flaps, both RSTLs and facial aesthetic subunit principles should be considered.^[15–17]^ In terms of the facial aesthetic unit principles, Gonzales-Ulloa described the original 14 facial aesthetic units including the forehead, right and left cheeks, nose, right and left upper lids, right and left lower lids, right and left ears, upper lip, lower lip, mental region, and neck.^[13]^ He believed that superior surgical results could be obtained in complex facial reconstructions by replacing lost skin with grafts or flaps of similar histology, thickness, and texture. As such, surgeons must attempt to hide the surgical margins within the natural borders of each facial unit.^[13]^ Menick et al revitalized interest in the field of facial aesthetic units by introducing the facial subunit theory (Figs. 5 and 6). They proposed that if a suture line is matched to the shape of a particular subunit, the natural appearances of light and shadow are restored, thereby allowing the reconstruction to be imperceptible because the scars are perceived as part of the normal facial topography.^[15]^ Until now, many local flap facial reconstruction techniques based on these concepts have been devised and reported.^[14,17]^ In this study, we categorized the location of the defects according to the facial aesthetic unit concept and considered them in the performance of KDPIF reconstructions (Table 1).

The KDPIF is a multi-perforator-based advancement flap designed to mimic the keystone in Roman arches (a curvilinear trapezoidal shape).^[3]^ As mentioned previously, this flap has been described as a combination of 2 opposing V-Y flaps. The initial V-Y advancement at the corners of the keystone flap along the longitudinal axis toward the center and parallel to the defect provides residual laxity within the flap. This orientation allows for translation or advancement of the keystone horizontally into the defect.^[3,18]^ Recently, the KDPIF has been widely used as both a primary and an alternative method in reconstructive surgery. Various studies have described the use of KDPIF to cover full-thickness cutaneous defects in various anatomical locations including the face, neck, trunk, and extremities.^[3,18–20]^

Behan et al described the reconstruction of large facial defects around the parotid gland using KDPIF in a series of 62 patients.^[5]^ These authors presented the KDPIF in accordance with the facial angiotome concept as a single-stage reconstructive option for covering parotid defects that were relatively easy to perform and obviated the need for free tissue transfer.^[5]^ They reported that KDPIF provided thin and pliable tissue that was well-matched in color and provided highly reliable and reproducible results.^[5]^

For small-to-moderate facial defects, several studies in the PubMed database primarily discussed nasal reconstructions followed by posterior auricular and medial canthal reconstructions.^[6–8,21,22]^ Previous studies have described KDPIF as a single-stage reconstructive option that provided sound functional and aesthetic outcomes for nasal defects up to 2 cm in diameter, and partial thickness alar defects up to 1.5 cm in diameter.^[6,7]^ Some of these studies have also reported that KDPIF nasal reconstructions were safe and reproducible even in the hands of inexperienced surgeons, albeit under guidance.^[6,7]^ It is important to consider the RSTL and facial aesthetic unit concepts, especially in nasal reconstructions because the nose is included in the central facial unit, and also has its own complex surfaces consisting of convex and concave regions. Therefore, we devised a design that would place the flap and incision lines within and along each nasal aesthetic subunit. The final results showed favorable aesthetic outcomes, with the skin creases mimicking the natural creases.^[16,23]^ As a result, the patient satisfaction scores were excellent (rated as 10/10) and the cosmetic outcomes resulted in excellent ratings by all 3 independent plastic surgeons in our 4 nasal reconstruction cases. In addition, we used Ω- variant type III KDPIF for both lateral side walls of the nose to cover moderate defects on the nasal dorsum. This promoted increased movement of the flap and decreased wound tension with less noticeable scars that were more parallel to the RSTLs. Thus, we successfully covered larger nasal defects than previously reported,^[6]^ with the largest defect size of 2.5 × 3 cm^2^ from the KDPIF described in Case 9.

To the best of our knowledge, few reports have focused on KDPIF reconstructions for small-to-moderate defects of other facial regions, such as the cheek, temple, and forehead. In the present study, we extended the KDPIF reconstruction for small-to-moderate facial defects to include not only the nose but the entire facial region. In the present study, we performed KDPIF reconstructions of these regions in 2 patients with temple defects (lateral subunit of the forehead unit), 4 with cheek defects (lateral subunit of the cheek unit), 1 with a preauricular defect (zygomatic subunit of the cheek unit), 3 with malar defects (medial subunit of the cheek unit), 1 with a nasolabial fold defect (medial subunit of the cheek unit), 1 with a defect in the area of the angle of the mandible, and 1 with a forehead defect (central subunit of the forehead unit). Although the peripheral facial units (cheek, forehead, and chin) gather a smaller focus of attention and tend to be of secondary visual interest,^[17,24]^ reconstructive surgeons should consider the aesthetic aspects of these areas and the patient's desire for minimal scarring. In this study, we successfully covered a 3 × 3.5 cm^2^-sized defect and attempted to move the flap posteriorly and laterally along the RSTL and the facial unit boundaries as much as possible, due to the off-center location of the peripheral facial unit reconstruction. In this way, the scars would be concealed by the natural skin creases. All patients were aesthetically pleased with their final outcomes. Among 13 patients, excepting the 1 with the nasal reconstruction, the average patient satisfaction score was 7.76 and the cosmetic outcomes resulted in fair to excellent ratings by 3 independent plastic surgeons.

We did not use a hand-held ultrasound Doppler device for detecting the perforators in any of our patients. We believe that it may be unnecessary to locate the perforators with either computed tomographic angiography or a hand-held ultrasound Doppler device in KDPIF reconstructions for small-to-moderate facial defects because the face is rich in perforators that have plenty of vascular connections resulting in reliable vascular perfusion. Furthermore, minimal undermining of the flap in KDPIF guarantees stable flap perfusion and vascularity.^[7]^ Thus, the KDPIF can survive successfully and without complication after facial reconstruction. Minimal undermining of the flap is an inner conceptual feature of KDPIF placement in comparison with conventional local and perforator flaps, resulting in shorter operative times, lower morbidity, and faster local recovery.^[7]^ However, to the best of our knowledge, no study has numerically quantified the extent of minimal undermining. In our cases, we performed undermining 3 to 4 mm from the flap margin following the release of the fibrous subcutaneous septa and deep fascia. None of the flaps in this study had compromised vascular perfusions and all survived fully without any complications.

Although we successfully achieved KDPIF reconstructions in small-to-moderate facial defects, our study has some limitations. The present study was a non-randomized retrospective clinical review with a comparatively small number of patients and no comparison groups. This inevitably resulted in selection and confounding biase. In the future, well-designed prospective studies including larger numbers of patients are required to ensure the consistency of favorable results. In addition, further studies should include whether the RSTL-oriented KDPIF reconstruction considering the facial aesthetic unit concept could be applied to large facial defects. However, it might be difficult to apply our technique (RSTL-oriented KDPIF reconstruction) to a defect that is not RSTL-oriented, and the final scars may not precisely correspond with RSTL in some defects. In such cases, we consider that the effort to move the flap posteriorly and laterally along the RSTL and facial unit boundaries as much as possible could yield good results as mentioned previously. In spite of these limitations, we believe that the KDPIF reconstruction is definitely a simple, safe, and reproducible option among local flap procedures for covering small-to-moderate facial defects.

## 7 Conclusions

We presented the RSTL-oriented KDPIF reconstruction technique in consideration of the facial aesthetic subunit concept for covering small-to-moderate defects in both central and peripheral facial units. The KDPIF technique has many advantages, including its simple defect-adaptive design, easy reproducibility, safety, and shortened procedure times resulting from minimal flap undermining and dissection. We also found that the RSTL-oriented KDPIF reconstruction techniques, in consideration of the facial aesthetic subunit concept, achieved ideal outcomes (replacement of “like-with-like”) without difficulty.^[3–7,13,14]^ We believe that the RSTL-oriented KDPIF reconstruction, considering the facial aesthetic subunit concept, may be a good alternative to conventional local and perforator flaps for covering small-to-moderate facial defects with superior aesthetic outcomes.

## Acknowledgments

We would like to thank Editage (www.editage.com) for English language editing and publication support.

## Author contributions

**Conceptualization:** Kyu Nam Kim.

**Data curation:** Hyo Bong Kim, Young Keun Kim, Hoon Kim.

**Formal analysis:** Chi Sun Yoon, Hyo Bong Kim, Young Keun Kim, Hoon Kim, Kyu Nam Kim.

**Investigation:** Chi Sun Yoon, Kyu Nam Kim.

**Methodology:** Kyu Nam Kim.

**Project administration:** Kyu Nam Kim.

**Resources:** Chi Sun Yoon, Hyo Bong Kim.

**Supervision:** Kyu Nam Kim.

**Visualization:** Kyu Nam Kim.

**Writing – original draft:** Chi Sun Yoon.

**Writing – review & editing:** Kyu Nam Kim.

## Supplementary Material

Supplemental Digital Content
