# Supplementary material for: Relaxed skin tension line-oriented keystone-designed perforator island flaps considering the facial aesthetic unit concept for the coverage of small to moderate facial defects
Source: Medicine (Baltimore). 2019 Jan 18;98(3):e14167. doi: 10.1097/MD.0000000000014167 (PMC6370060; doi:10.1097/MD.0000000000014167)
Supplement: Supplemental Digital Content [file medi-98-e14167-s001.docx]

**Table S1.** Postoperative cosmetic outcomes: plastic surgeon’s global impression of changes according to the Harris 4-stage scale and postoperative patient-satisfaction surveys

| **Case** | **PS1** | **PS2** | **PS3** | **Patient satisfaction score** |
| --- | --- | --- | --- | --- |
| 1 | Good | Excellent | Good | 8 |
| 2 | Good | Good | Fair | 7 |
| 3 | Excellent | Excellent | Good | 9 |
| 4 | Good | Fair | Good | 7 |
| 5 | Good | Excellent | Good | 7 |
| 6 | Excellent | Excellent | Excellent | 10 |
| 7 | Excellent | Excellent | Good | 9 |
| 8 | Excellent | Excellent | Excellent | 10 |
| 9 | Excellent | Excellent | Excellent | 10 |
| 10 | Good | Excellent | Good | 8 |
| 11 | Excellent | Good | Good | 9 |
| 12 | Good | Fair | Good | 8 |
| 13 | Good | Fair | Fair | 7 |
| 14 | Excellent | Excellent | Excellent | 10 |
| 15 | Good | Good | Good | 7 |
| 16 | Good | Good | Excellent | 8 |
| 17 | Good | Good | Good | 7 |

PS: plastic surgeon
